# Teleworking in the Covid-19 Pandemic: The Effects of Life-Work Conflict on Job Outcomes and the Role of the IT Telework Environment

**DOI:** 10.1007/s12599-023-00800-3

**Published:** 2023-03-17

**Authors:** Christoph Weinert, Tim Weitzel

**Affiliations:** University of Bamberg, An der Weberei 5, 96049 Bamberg, Germany

**Keywords:** Teleworking, Work from home, Life-work conflict, Work-home conflict, Role theory, Routine and innovative job performance, Work exhaustion, Job satisfaction

## Abstract

Teleworkers who live and work in the same space are vulnerable to conflicts between personal life and work (LWC). The Covid-19 lockdowns increased the intensity and risk of LWC and changed telework conditions, confronting teleworkers with difficult personal situations and often ill-equipped telework environments. To develop a better understanding of the effects of different LWC dimensions (e.g., time, strain, behavior) on work exhaustion, job satisfaction, routine and innovative job performance and the role of the IT telework environment among teleworkers in the Covid-19 pandemic, a research model based on a sample of 249 teleworkers was developed and validated. The findings show that LWC has adverse effects on job outcomes and that the IT telework environment moderates these effects. The study contributes to the telework and role conflict literature by revealing the essential role of the IT telework environment and by differentiating between routine and innovative job performance among teleworkers.

**Supplementary Information:**

The online version contains supplementary material available at 10.1007/s12599-023-00800-3.

## Introduction

Over the years, work has been shifting from traditional work arrangements (e.g., office work) to modern work arrangements, such as teleworking (Allen et al. 2015). At-home teleworkers (hereafter teleworkers) are self-employed contractors or employees of an organization who regularly work for an organization from home at least some of the time (Lindström et al. 1997). This work arrangement requires teleworkers to execute work tasks from home using various information technologies (IT) (Bailey and Kurland 2002). Teleworking offers employees and organizations several potential advantages, such as greater flexibility, autonomy, job satisfaction, productivity and employee morale, as well as lower turnover rates (Kuruzovich et al. 2021; Bélanger 1999; Bélanger et al. 2001; Kossek et al. 2006; Mazmanian et al. 2013).

However, despite these potential advantages of teleworking, scholars have shown that some teleworkers are more vulnerable to role conflicts in comparison to office-worker colleagues (Delanoeije et al. 2019; Greer and Payne 2014). According to role theory (Ashforth 2001), individuals adopt multiple roles which can interfere with each other (Greenhaus and Beutell 1985). For example, in their personal lives, individuals may have roles as parents, siblings, spouses, friends or caregivers, which may interfere with the roles they may have at work, such as colleagues and supervisors. This interference is called personal life-work conflict (LWC)^1^ (Greenhaus and Beutell 1985). If personal life roles interfere with work roles, LWC can negatively influence job outcomes (Venkatesh et al. 2019; Bélanger et al. 2001), such that employees are more exhausted from work, less satisfied at work (Venkatesh et al. 2019), and exhibit lower job performance (Sarker et al. 2018; Bélanger et al. 2001). LWC is more common among teleworkers than it is among office workers since working at home weakens the boundaries between personal life roles and work roles and because boundary-crossing activities are more common (Delanoeije et al. 2019; Greer and Payne 2014). Teleworkers switch between personal life roles and work roles more frequently and more quickly than office workers (Matthews et al. 2010; Delanoeije et al. 2019), and their roles are much less clearly defined by contextual clues (Raghuram and Wiesenfeld 2004). For example, teleworkers often lack an office workplace context that helps define their work roles. Besides, teleworkers working at home do not commute, which often serves as an adaptation phase between personal and work roles (Jachimowicz et al. 2021).

The Covid-19 pandemic accelerated the shift from traditional work arrangements (e.g., office work) to teleworking. The worldwide outbreak of Covid-19 led governments and companies to force or strongly encourage many more employees than usual to work from home. Before the Covid-19 pandemic, approximately five percent of employed people in the European Union (EU) worked from home (Milasi et al. 2020). The share of teleworking reached a peak of 40 percent and is expected to remain high in the post-Covid-19 pandemic (Eurofound 2020). Interference between personal life and work has been identified as a key telework challenge during the Covid-19 pandemic (Wang et al. 2020). The Covid-19 pandemic accelerated these boundary-crossing activities and hence LWC.

The Covid-19 pandemic has changed teleworking conditions, forcing teleworkers to deal with complex personal situations and work in a less-than-ideal telework environment (Carillo et al. 2021). Due to unpredictable waves of infection and social contact restrictions, teleworkers face complex personal situations, at times potentially including caring for children at home when schools and kindergartens are closed, potentially sharing workspace with children, partners, or spouses also working from home. Temporary curfews and contact restrictions create additional complexity, often requiring teleworkers to use the same space for work and personal life, which creates interference and may have to shift quickly and frequently between personal life roles and work roles, posing the risk of inappropriately carrying over behaviors from personal life roles to work roles. At the same time, especially during the Covid-19 lockdowns, teleworkers often lack sufficient IT, including hardware, software and internet capability (Carillo et al. 2021). For example, more than half of Germany’s teleworkers report lacking sufficient IT equipment during the first year of the Covid-19 pandemic (Statista 2022). Some teleworkers report working on small laptops at the kitchen table and having limited access to organizational software and an insufficient internet connection during Covid-19 lockdowns. Extant research also reveals that a telework environment with sufficient IT promotes productive teleworking (He et al. 2021; Bélanger et al. 2001).

Especially since the outbreak of Covid-19, organizations have been dependent on new working arrangements such as teleworking to stay productive. They strive to minimize personal life-work conflicts that arise when teleworkers use the same space for work and personal life, and that could lead to undesirable job outcomes by providing an IT telework environment that maximizes work productivity and satisfaction. To support these aims, our study examines LWC and its influence on job outcomes among teleworkers, focusing on teleworking from home during the Covid-19 pandemic. In addition, we investigate the role of the IT telework environment. We ask the following research question:

*How does LWC influence job outcomes among teleworkers and how does the IT telework environment influence this relationship in the Covid-19 pandemic?*

**Fig. 1:**
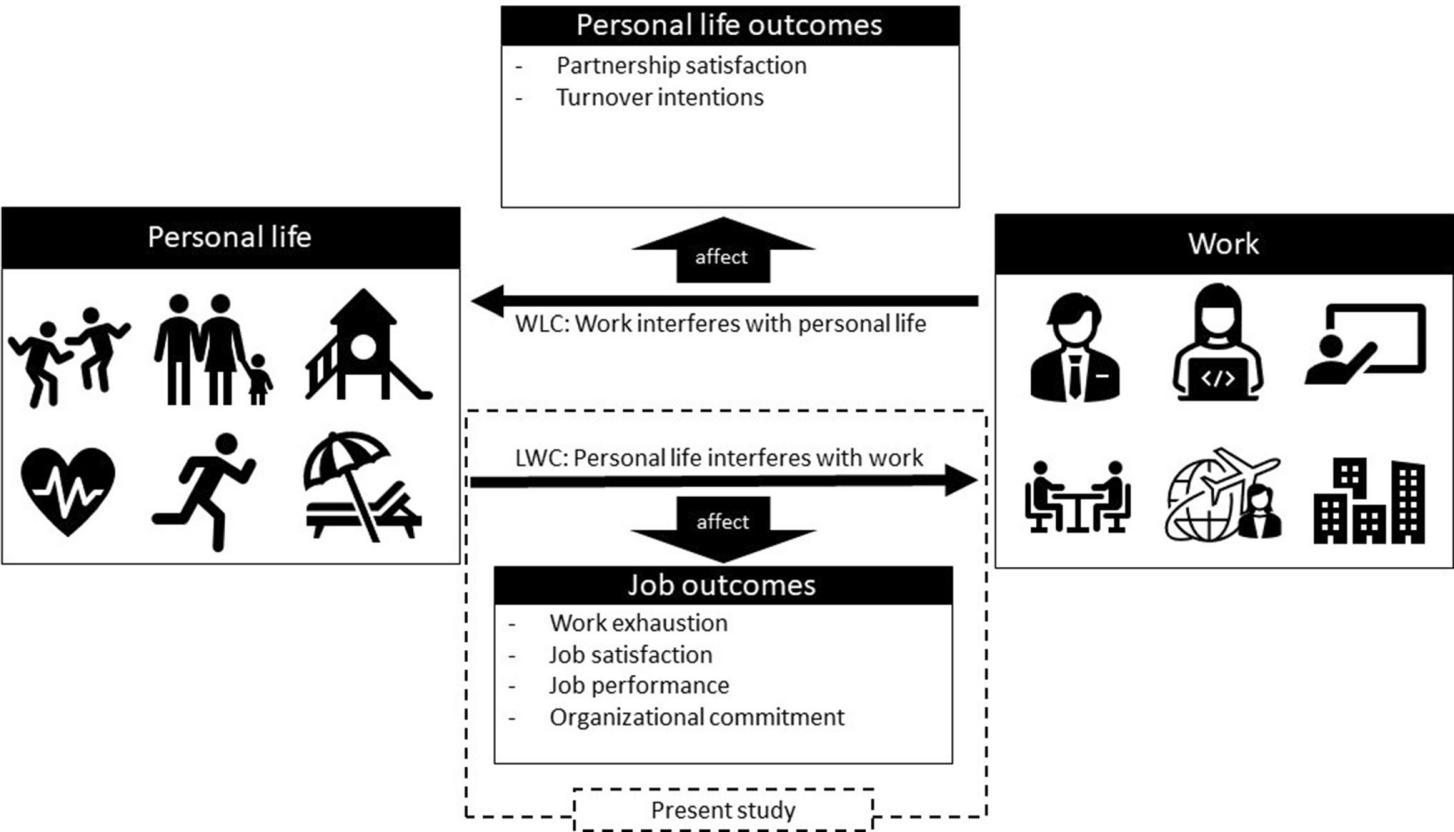
WLC and outcomes, LWC and outcomes, and focus of the study

To answer this research question, we draw on the role theory (Ashforth 2001). In our study, we refer to the interference of work roles with personal life roles as work-personal life conflict (WLC), and the interference of personal life roles with work roles as personal life-work conflict (LWC)(Greenhaus and Beutell 1985). WLC negatively influences one’s personal life roles, whereas LWC negatively influences one’s work roles (ten Brummelhuis and Bakker 2012; see Fig. 1). Our study focuses solely on LWC. We look at the outcomes in the work role by investigating whether and how LWC negatively impacts job performance and satisfaction. Following the lead of extant literature, we differentiate between time-based LWC (T-LWC), strain-based LWC (S-LWC), and behavior-based LWC (B-LWC) (Venkatesh et al. 2019) and investigate their effects on three main job outcomes in the LWC and teleworking context – work exhaustion, job satisfaction, and job performance (e.g., routine and innovative) (Venkatesh et al. 2019; Sarker et al. 2018), as well as the role of the IT telework environment. To test our hypotheses, we conducted an online survey with a sample size of 249 teleworkers and analyzed the data taking a partial least squares structural equation modeling (PLS-SEM) approach. Our results demonstrate that LWC has adverse effects on job outcomes. Routine job performance is only negatively influenced by T-LWC. Work exhaustion, job satisfaction, and the IT telework environment positively affect routine job performance. In contrast, innovative job performance is reduced by B-LWC and work exhaustion and is increased by job satisfaction. The IT telework environment has no direct effect on innovative job performance but reduces the effect of LWC on innovative job performance. Our results contribute to telework and LWC research by showing that a well-suited IT telework environment is essential for telework job performance and that its mediating effect depends on the routine or innovative aspect of job performance. Our study focuses on LWC and job outcomes in the work role. It thereby sheds new and differentiated light on how personal life-work conflicts among teleworkers affect job outcomes and on the degree to which the IT telework environment mediates those effects.

The remainder of this paper is organized as follows. First, we explain the theory and dimensions of role conflicts. Then, we outline relevant research into telework and role conflicts. Based on these theoretical foundations, we develop hypotheses. We subsequently explain our methodology and results. Lastly, we discuss the theoretical and practical contributions of our study.

## Theoretical Background

In this section, we provide an overview of the role conflict phenomenon, discuss the specifics of the teleworking context and summarize relevant extant research into LWC and WLC.

### Role Conflict: Work-Personal Life and Personal Life-Work

Role conflict is based on the role theory developed by Ashforth (2001). According to role theory, a role is a unique set of behaviors, requirements, responsibilities, and identities (Ashforth 2001). Individuals can act in different roles, such as employees in a work role, or as fathers or friends in a personal life role. Each role is associated with its own objectives, beliefs, values, norms, interaction styles, and time horizon (Ashforth 2001).

Role conflict can be understood as *“a form of interrole conflict in which the role pressures from the work and [personal life] domains are mutually incompatible in some respect”* (Greenhaus and Beutell 1985, p. 77). Role conflicts are bi-directional (Gutek et al. 1991). In the context of our study, the interference (or detrimental effect) of work on personal life is called work-personal life conflict (WLC), and the interference of personal life on work is called personal life-work conflict (LWC) (Carlson et al. 2000; Greenhaus and Beutell 1985). LWC negatively affects employees in their working roles, whereas WLC leads to negative effects on their personal life roles (Brummelhuis and Bakker 2012). To better understand potentially undesirable job outcomes of teleworkers undergoing a rapid and unexpected transition from traditional office work to telework, we need to understand how working from home leads to greater conflict between their personal life roles and their work roles. For this reason, we focus on LWC (see Fig. 1).

LWC can be classified into three dimensions: time-based, strain-based, and behavior-based LWC (Greenhaus and Beutell 1985). *Time-based LWC (T-LWC)* occurs when different roles (e.g., personal life vs. work) compete for one’s time. Logically, the time spent within one role (e.g., personal life role) cannot be spent within another role (e.g., work role). This form of LWC is associated with excessive work time and schedule conflict, role overload, and time pressure. *Strain-based LWC (S-LWC)* occurs when symptoms such as tension, anxiety, fatigue, and depression arising in one role limit one’s ability to meet the responsibilities of another role. *Behavior-based LWC (B-LWC)* occurs when the behavior performed in one role is incompatible with the expectations regarding the behavior in the other role. Past literature characterizes this conflict by the expectation that behavior in the work role is rather self-reliant, emotionally stable, aggressive, and objective. In contrast, behavior in the personal life role is warm, nurturing, emotional, and vulnerable (Greenhaus and Beutell 1985). B-LWC occurs when individuals are unable to adjust behaviors performed in their personal life roles to the behaviors they are expected to perform in their work roles (Greenhaus and Beutell 1985; Carlson et al. 2000).

### At-Home Teleworking

Early information systems (IS) literature offers various classifications of telework depending on the use of IT and where the work is done (Lindström et al. 1997). This study focuses on at-home teleworking (also called work at home) to reflect the increase of employees working from home in the (post)-Covid-19 pandemic.^2^ We define at-home teleworkers (hereafter teleworkers) as self-employed people and employees of an organization who work from home to at least some extent (Lindström et al. 1997). Due to recent rapid IT developments, such as inexpensive and reliable broadband communication, cloud computing, and software as a service, the way individuals work and live has changed. These changes have enabled employees to work away from the core organization at home using IT (Bailey and Kurland 2002). Teleworking has become a viable alternative to traditional office-based work for many job types (van der Meulen et al. 2019). Teleworking provides organizations with new ways of doing work and structuring the organization. Early research on telework looks at the positive side of teleworking, exploring what makes the teleworking day easier or more difficult (Suomi and Pekkola 1998; Guimaraes and Dallow 1999) or its advantages, such as flexibility and autonomy (Kossek et al. 2006). Most extant literature concentrates on job outcomes such as turnover intention (Igbaria and Guimaraes 1999), job satisfaction (Igbaria and Guimaraes 1993), knowledge sharing (Bélanger and Allport 2008), or job performance (Bélanger et al. 2001; van der Meulen et al. 2019).

Recent research into teleworking during the Covid-19 lockdowns has focused on how employees adjusted to the rapid and unexpected change from office work to telework, exploring the influence of crisis-specific variables such as professional isolation, telework environment, workload increase, and stress on job outcomes (Carillo et al. 2021). Scholars have also investigated the social and technical aspects of teleworking and their effect on job outcomes during the pandemic. Research has shown that due to rapid changes in teleworking, environmental affordances are withdrawn and that more features of technological affordances are used to facilitate work and social interactions. Technology affordance reduces isolation and facilitates career advancement among teleworkers but negatively affects communication (Waizenegger et al. 2020). In an academic context, family-work conflicts worsen teaching staff’s attitudes toward work from home, whereas perceived IT usefulness improves it. In turn, a positive attitude toward work from home increases work productivity (AbuJarour et al. 2021). Research into changes in social interaction among employees making the rapid and unexpected shift from office work to teleworker during the Covid-19 pandemic yielded mixed results. Teleworkers report finding it difficult to maintain social interaction via IT, but also perceive social interaction in the workplace as a distraction (Lal et al. 2021). Studies find that IT use and IS quality, including for social exchange, influence job outcomes such as job satisfaction and performance (Kuruzovich et al. 2021). Teleworkers report having to invest more effort in communication when working at home than when working in an office and report needing new skills to build a sense of belonging to the team and the work itself. Such “belonging through technology” influences teleworkers’ well-being and productivity (Hafermalz and Riemer 2021).

### Relevant Extant Research into Role Conflict and its Outcomes

IS scholars have investigated the conflict between work and personal life roles in several contexts. Various examinations focus on WLC and IS professionals (Ahuja et al. 2007; Armstrong et al. 2015; Sarker et al. 2010), technostress (Ayyagari et al. 2011), and technology addiction (Turel et al. 2011). Research focuses on the antecedents of WLC and shows that factors such as time difference, the frequency of communication, and the number of distributed locations influence WLC (Sarker et al. 2010). In the technostress and addiction context, research reveals presenteeism (Ayyagari et al. 2011) and technology addiction (Turel et al. 2011) as antecedents of WLC. In the consumer IT context, results show that if office employees may not use consumer IT products that adhere to their role segmentation preference for work purposes, they perceive high levels of WLC (Köffer et al. 2014, 2015). Moreover, IS scholars investigate the effects of WLC on different consequences such as organizational commitment (Ahuja et al. 2007), work exhaustion (Ahuja et al. 2007; Armstrong et al. 2015; Weinert et al. 2014, 2015, 2016), turnover intention (Ahuja et al. 2007; Sarker et al. 2018), and performance (Sarker et al. 2018).

Relatively few studies focus on LWC in contexts such as decision making, consumer IT, technology addiction, and productivity. Related literature focuses mostly on the consequences of LWC. For example, research demonstrates that LWC reduces the ability of chief executive officers to make decisions for the organization, which negatively affects overall company performance (Reina et al. 2017). In the context of technology addiction among children, LWC influences job outcomes such as job satisfaction, organizational commitment, and work exhaustion (Venkatesh et al. 2019). Also, LWC negatively influences employees' attitudes toward working from home, which in turn, increases productivity (AbuJarour et al. 2021).

As illustrated in Table 1, most extant IS literature concentrates on WLC. Only a few studies consider the effects of LWC, whereby the consequences are more critical for organizations because employees whose personal life roles interfere with their work roles often demonstrate undesirable job outcomes in the working role. In addition, only a few studies differentiate among the various dimensions of LWC (e.g., time-, strain-, behavior-based) and its effect on job outcomes (Venkatesh et al. 2019). Moreover, most extant IS literature focuses on IS professionals (Ahuja et al. 2007; Sarker et al. 2010, 2018), individuals stressed by IT (Ayyagari et al. 2011) or individuals addicted to IT (Turel et al. 2011; Venkatesh et al. 2019). There is a clear gap in research into the effects of LWC among at-home teleworkers, even though this share of the workforce not only increased rapidly due to the Covid-19 pandemic, but is also particularly susceptible to role interference (Greer and Payne 2014; Delanoeije et al. 2019). During Covid-19 lockdowns, many teleworkers needed to switch between personal life roles and activities and work roles and activities several times a day, for example, by interrupting their work to tend to the needs of family members or loved ones. The absence of boundaries between the different roles increases the potential for conflict between personal life roles and work roles, resulting in undesirable job outcomes (Greer and Payne 2014). Employees who were forced or strongly encouraged to switch from office work to telework unexpectedly and rapidly due to Covid-19 pandemic restrictions often initially worked under poor IT conditions and lacked sufficient IT tools (Carillo et al. 2021). Past literature provides evidence that having sufficient IT conditions and access to professional IT tools at home increases teleworkers’ productivity when working from home (He et al. 2021; Bélanger et al. 2001). To fill the abovementioned research gaps, this study concentrates on two effects: Firstly, we focus on the main dimensions of LWC and their influence on work exhaustion, job satisfaction, and job performance; secondly, we investigate whether the IT telework environment is a prerequisite for job performance among the vulnerable group of teleworkers during the Covid-19 pandemic.

**Table 1:** Overview of role conflict

Author	Role conflict (WLC/LWC)	Dimensions (time, strain, behavior)	Context	Job outcomes
Ahuja et al. (2007)	WLC	–	IS professionals	Work exhaustion Organizational commitment Turnover intention
Sarker et al. (2010)	WLC	–	Distributed work arrangement GSD or IT professionals	–
Ayyagari et al. (2011)	WLC	–	Technostress	Strain (techno-exhaustion)
Turel et al. (2011)	WLC	–	Addiction to mobile email	Organizational commitment
Armstrong et al. (2015)	WLC	–	Career-Family conflict IS careers IS professionals	Exhaustion from IS career experience Affective commitment to IS profession Turn-away intention
Köffer et al. (2014, 2015)	WLC	–	Privately-owned IT	–
Weinert et al. (2016)	WLC	Time, strain, behavior	IT usage Exhaustion	Techno-exhaustion Work exhaustion
Reina et al. (2017)	LWC	–	CEO family-to-work conflict Firm performance	Decision-making comprehensiveness Firm performance
Sarker et al. (2018)	WLC	–	GSD or IT professionals Work-personal life conflict	Turnover intention Performance
Venkatesh et al. (2019)	LWC	Time, strain, behavior	Internet addiction of children Parting behavior	Job satisfaction Organizational commitment Work exhaustion
AbuJarour et al. (2021)	LWC	–	Academic work	Productivity

## Hypotheses Development

**Fig. 2:**
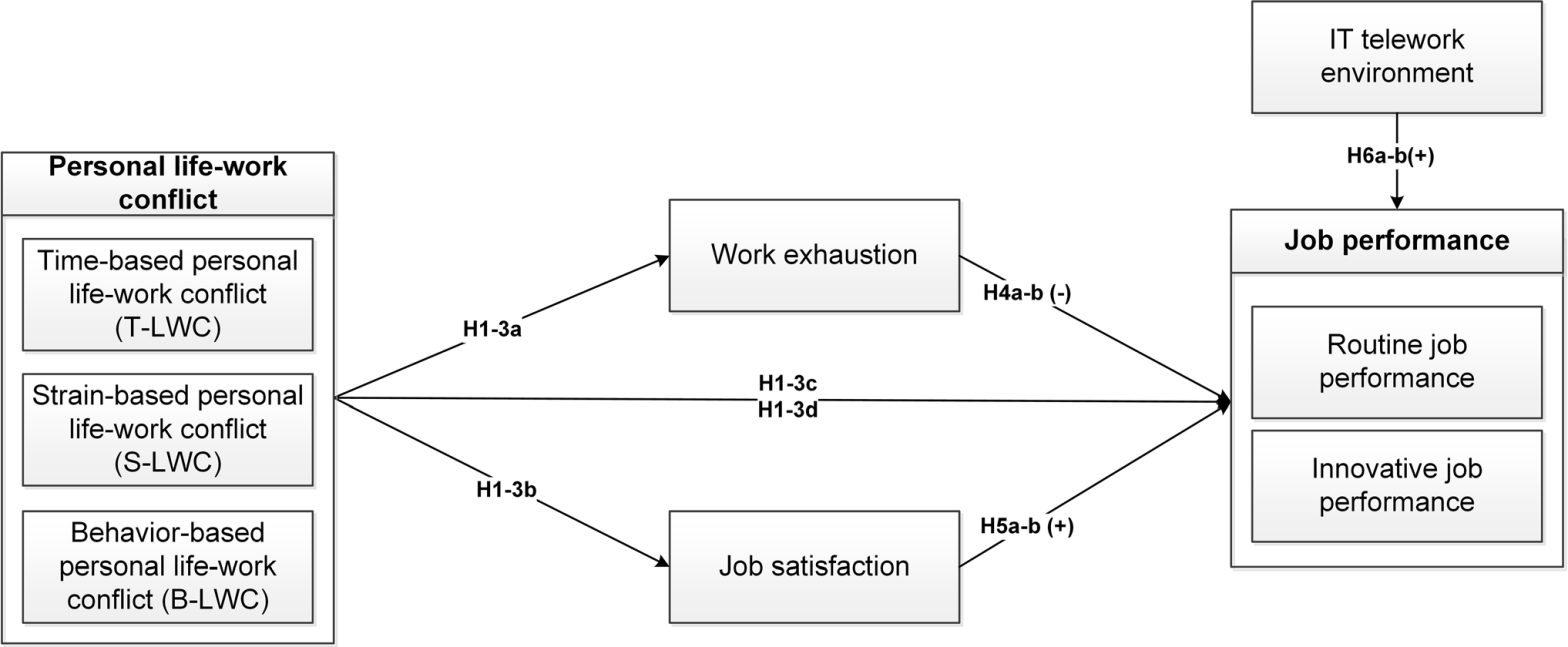
Research model

In investigating the effects of the interference between personal life and work and their consequences on telework, we focus on three job outcomes that research has identified as relevant in the role conflict and telework context: work exhaustion (Venkatesh et al. 2019; Ahuja et al. 2007; Wang et al. 2020), job satisfaction (Venkatesh et al. 2019; Kuruzovich et al. 2021; Wang et al. 2020) and job performance (Kuruzovich et al. 2021; Wang et al. 2020). Work exhaustion is defined as the depletion of mental resources when accomplishing work goals (Ahuja et al. 2007). Job satisfaction is defined as *“a pleasurable or positive emotional state resulting from the appraisal of one’s job or job experiences”* (Venkatesh et al. 2019, p. 910). Following previous research, we differentiate between two aspects of job performance: routine and innovative job performance (Ali-Hassan et al. 2015; Janssen and Van Yperen 2004; Katz 1964). Traditionally, many scholars assumed that telework involved mainly routine tasks rather than knowledge tasks involving creativity and innovation (Pérez Pérez et al. 2002). Routine job performance refers to executing job tasks consistently as required by the job description, while innovative job performance refers to undertaking activities that go beyond the job requirements to obtain new and beneficial outcomes (Ali-Hassan et al. 2015; Janssen and Van Yperen 2004). Due to the Covid-19 situation, teleworkers were expected to perform the full range of tasks while working from home, often in a less-than-ideal IT environment (i.e., under poor IT conditions and lacking full access to professional IT tools) (cf. Carillo et al. 2021) (Fig. 2).

Teleworkers who perceive strong LWC are more likely to experience the depletion of mental resources (Venkatesh et al. 2019; Wang et al. 2020). T-LWC occurs when time spent in personal life roles cannot be spent in work roles such that the duties in this role cannot be accomplished. During the Covid-19 lockdowns, many teleworkers had to allocate more than usual time to personal life roles to attend to the needs of the family or loved ones, leaving less time than usual for work roles (Holland et al. 2016; Wheatley 2012). Besides, as time falls short in the work role, teleworkers experience work-related time pressure and time urgency (Greenhaus and Beutell 1985), which increases work exhaustion (Eckhardt et al. 2012). In other words, if time is spent on activities in personal life, this time cannot be used for work-related tasks. Therefore, we hypothesize that when teleworkers have less time for their work tasks, more emotional resources are required, and they experience increased fatigue at work (H1a). LWC also affects job satisfaction (Venkatesh et al. 2019; Wang et al. 2020). We hypothesize that teleworkers may be more prone to allocate more time during the day to their personal life roles than office workers, leading teleworkers to work under time pressure (Greenhaus and Beutell 1985), resulting in negative emotional states and lower job satisfaction (Gerich and Weber 2020) (H1b). The interference of personal life roles with work roles is critical for organizations as the consequences decrease personal job outcomes of teleworkers. WLC influences personal life factors and job outcomes, leading to more failures (Lapierre et al. 2012) and thereby lowering routine job performance. This conflict between personal life roles and work roles further hinders work task performance because the boundaries between the roles are weakened when working from home (Greer and Payne 2014). We hypothesize that since time spent in personal life roles logically cannot be spent in work roles, there may be insufficient time to complete all work tasks on time, potentially leading to missed deadlines and delayed routine task completion (H1c). Innovation is a complex and challenging cognitive and social activity. Innovative job performance involves developing and applying something new for which the knowledge and strategies have not yet been mastered (Janssen and Van Yperen 2004). Deep processing and new knowledge are needed to achieve innovative job performance (Elliot and McGregor 2001). As discussed above, many teleworkers perceive T-LWC and allocate less time to their work roles. Since innovation is a complex task that needs sufficient time, we assume that T-LWC reduces innovative job performance (H1d).

### H1

The higher the T-LWC, **(a)** the higher the work exhaustion, **(b)** the lower the job satisfaction, **(c)** the lower the routine job performance and **(d)** the lower the innovative job performance among teleworkers in the Covid-19 pandemic.

According to the conservation of resources (COR) theory (Hobfoll et al. 2018), individuals aim to acquire and maintain resources. As especially evident during Covid-19 lockdowns, many teleworkers allocate more emotional resources to their personal life roles because they are in the home environment, potentially with partners, spouses, parents, children, roommates, etc. In general, tensions occurring in personal life roles due to childcare or demands from partners and friends consume emotional resources, making them unavailable in the work role. In other words, the allocation of resources (e.g., energy) to personal life roles led to fewer resources (e.g., energy) for work roles. As the depletion of resources leads to emotional exhaustion (Hobfoll and Shirom 2001), we hypothesize that S-LWC increases work exhaustion (H2a). During Covid-19 lockdowns, attending to challenging personal life duties made many teleworkers feel tired and drained, leaving them unable to perform well on the job, causing negative emotional states and reduced job satisfaction. According to expectation-confirmation theory (Oliver 1980), satisfaction is higher when expectations are confirmed and lower when they are not. Therefore, we hypothesize that job satisfaction is lower when job fulfillment expectations cannot be met due to negative emotional states related to challenging personal life responsibilities (H2b). Tensions experienced in personal life roles can also prevent teleworkers from performing routine work tasks adequately. Again applying COR theory (Hobfoll et al. 2018), resources consumed in personal life roles are not available in work roles, leaving teleworkers insufficient resources to perform routine work tasks effectively. As especially evident during the Covid-19 lockdowns, we hypothesize that teleworkers with childcare or other family or social responsibilities feel exhausted and have fewer resources available to accomplish work tasks (H2c). We assume that this phenomenon applies even more strongly to innovative job performance. As evident during the Covid-19 lockdowns, teleworkers had to allocate more resources to their personal life roles, leaving insufficient resources to perform the challenging and complex cognitive task of creating new knowledge and innovation. We hypothesize that teleworkers with low resources are ineffective in performing complex cognitive tasks to create new knowledge (H2d).

### H2

The higher the S-LWC, **(a)** the higher the work exhaustion, **(b)** the lower the job satisfaction, **(c)** the lower the routine job performance and **(d)** the lower the innovative job performance among teleworkers in the Covid-19 pandemic.

B-LWC arises when behaviors in personal life roles interfere with behaviors in the work role. While work role behavior is typically characterized by self-reliance, emotional stability, aggressiveness, and objectivity (Greenhaus and Beutell 1985), personal life role behavior is typically characterized by warmth, emotional transparency, vulnerability and subjectivity (Greenhaus and Beutell 1985). As especially evident during Covid-19 lockdowns, teleworkers who live and work in the same space, potentially alongside children, parents, partners and/or roommates, have to switch and adjust their behavior between their personal life roles and their work roles far more frequently than office workers (Delanoeije et al. 2019; Greer and Payne 2014). Frequent switching and adjusting require more emotional resources than usual, which, we hypothesize, leads to work exhaustion (H3a). Research indicates that when teleworkers inappropriately carry over personal life role behaviors to their work roles, they experience lower job satisfaction (Bruck et al. 2002) and appraise their job experience as negative and their behavior as embarrassing or weak. For example, when teleworkers interact with a child, partner or a friend at home and then quickly and frequently switch to interacting with an employee, coworker or supervisor, their personal life behavior, such as tone of voice, level of informality, colloquialisms, overly relaxed attitude, or degree of intimacy or vulnerability, may inappropriately spill over to their work behavior. We hypothesize that such B-LWC leads teleworkers to feel unsatisfied with their job (H3b). Inappropriate behavior in work roles also lowers routine job performance. For example, when teleworkers behave emotionally and vulnerably in work roles, their instructions may not be followed well or their requests prioritized appropriately, which, we hypothesize, lowers their job effectiveness (H3c). Finally, if teleworkers are not able to adjust their behavior to the work role (Greenhaus and Beutell 1985; Carlson et al. 2000) quickly or well enough, they cannot work objectively and focus on performing complex tasks to create new insights. Behaviors that are effective and necessary for teleworkers in their personal life roles may be ineffective in creating new knowledge or innovation (H3d).

### H3

The higher the B-LWC, **(a)** the higher the work exhaustion, **(b)** the lower the job satisfaction, **(c)** the lower the routine job performance and **(d)** the lower the innovative job performance among teleworkers in the Covid-19 pandemic.

Extant research shows that work exhaustion negatively affects job performance in general (Chilton et al. 2005; Cropanzano et al. 2003; Chen and Karahanna 2018). In our study, we differentiate between routine and innovative performance, assuming that routine job performance increases when teleworkers are exhausted. As evident during Covid-19 lockdowns, teleworkers with limited resources feel pressured to accomplish at least their routine tasks. In general, teleworkers report perceiving scrutiny from their supervisors and high personal performance expectations (Allsop et al. 2017). According to Parkinson’s Law (Parkinson 1957), performance pressure is associated with higher job performance (Eckhardt et al. 2012). In contrast, we assume that teleworkers exhausted from their routine work duties may lack the resources needed to create knowledge or innovation. This is supported by COR theory (Hobfoll et al. 2018), according to which work exhaustion depletes teleworkers’ resources, leaving insufficient resources to accomplish complex tasks to create novel and useful outcomes (Halbesleben and Bowler 2007) (H4d).

### H4

The higher the work exhaustion, **(a)** the higher the routine job performance and **(b)** the lower the innovative job performance among teleworkers in the Covid-19 pandemic.

Extensive psychological research has been dedicated to understanding the relationship between job satisfaction and job performance (see Judge et al. 2001 for an overview). Early research into human relations relates higher job satisfaction to better morale and hence better job performance (Strauss 1968) and later empirical studies in the IS field confirm that job satisfaction positively affects job performance (Tarafdar et al. 2010). Teleworkers who are happy at work and associate positive emotions with their job experience will accomplish their routine tasks effectively. This underscores fundamental behavioral theories positing that a favorable attitude or positive evaluation results in desirable behavior (Fishbein and Ajzen 1975; Ajzen 1991) (H5a). The same applies to the relationship between job satisfaction and innovative job performance. Previous telework research reveals that higher job satisfaction leads to better innovative job performance (Bysted 2013). Therefore, we hypothesize that teleworkers with a positive emotional state due to higher job satisfaction are more motivated and better able to perform complex tasks and develop new knowledge and innovation than teleworkers in a negative emotional state due to lower job satisfaction (H5b).

### H5

The higher job satisfaction, **(a)** the higher the routine job performance, and **(b)** the higher the innovative job performance among teleworkers in the Covid-19 pandemic.

Early research into teleworking demonstrates that IT telework conditions, including the availability and suitability of IS and communication technology, increase the job performance of teleworkers (Bélanger et al. 2001). A recent investigation provides initial evidence that IS quality improved job outcomes, such as job performance among teleworkers during the first year of the Covid-19 pandemic (Kuruzovich et al. 2021). Due to the rapid and unexpected shift to telework during the Covid-19 lockdowns, many new teleworkers were initially ill-equipped (Carillo et al. 2021). Practical guidelines for post-Covid-19 teleworking suggest that sufficient IT equipment and tools are needed for teleworkers to avoid negative consequences for their performance (ILO 2020). In general, IS research shows that work tasks have been increasingly permeated by IT and IS applications over the past years (Richter et al. 2018; Richter 2020). For many telework jobs and tasks specifically, poor IT conditions, such as equipment and internet capability, and limited access to key IS applications lead to worse job performance, less consistent work quality and lower dependability (Bélanger et al. 2001; Kuruzovich et al. 2021). For example, without access to the video conferencing system or a reliable internet connection, teleworkers may be unable to complete work tasks efficiently during their assigned hours or complete such tasks on time. Therefore we hypothesize that a good IT telework environment is necessary for teleworkers to fulfill their job obligations, facilitating a higher routine job performance (H6a). To perform complex tasks of knowledge creation, teleworkers generate ideas with others, adapt ideas from others and build coalitions with others (Ali-Hassan et al. 2015). Past literature emphasizes that communication and exchange with others positively influence innovative job performance (Zheng 2010). As evident during the Covid-19 lockdowns, teleworkers require a good IT telework environment to facilitate collaboration with coworkers and supervisors (Waizenegger et al. 2020). Therefore, we hypothesize that a good IT telework environment leads to better innovative job performance among teleworkers (H6b).

### H6

The more sufficient the IT telework environment, (**a**) the higher the routine job performance and (**b**) the higher the innovative job performance among teleworkers in the Covid-19 pandemic.

## Methodology

To validate our model, we focused on teleworkers in different occupations using IT to accomplish their work tasks. We followed a crowdsourcing sampling strategy using Amazon Mechanical Turk (mTurk), which has been suggested as a valid and relevant sampling strategy (Steelman et al. 2014). Many consider it equivalent to other sampling strategies (Lowry et al. 2016). To overcome the limitations of online panels, such as attentiveness (Lowry et al. 2016), we followed previous researchers (Maier et al. 2019; Pirkkalainen et al. 2019) and used reverse-coding, randomized order of items and safety check questions (e.g., “I use IS for my work,” “I am employed,” “Please indicate your highest educational level,” “Please click on strongly disagree”). We received 467 responses. We excluded several responses because the participants did not pass the attention checks or did not fulfill the criteria to participate in the study. Our final sample encompasses 249 participants. All participants in our sample completed the survey and fulfilled the following criteria to participate. They are all teleworkers who have a current part- or full-time contract from an organization, have a job outside of mTurk, earn less than 25% of their monthly income through mTurk and use IT to conduct their daily work. The study was conducted in April 2021, and all participants were located in the United States. The respondents are a suitable data sample for our research because all participants currently have jobs and are between 25 and 54 years old. Sixty-eight percent of our survey participants have a college or university degree and have been working 31.2 months (2.6 years) at their current organization. 18.1 percent of our participants live alone and 81.9 percent live together with a partner. The majority of the participants have one to two children living at home (Table 2). The participants work for organizations in the engineering/IT/data process (21.1%), finance (16.9%), or communication sector (18.6%) and work in the field of finance/accounting/controlling (14.8%), IT (28.7%), or engineering (15.6%) (Table 7 in the Appendix, available online via 10.1007/s12599-023-00800-3).

**Table 2:** Study participants

Demographic (N = 249)			Work situation (in %)		Marital status (in %)		Education (in %)		Family situation (in %)		
Gender (in %) M = 1.49 SD = 0.501	Men (1)	51.5	Full-time contract	81.9	Single	13.2	Less than high school	3.8	Living situation (in %) M = 1.82 SD = 0.386	Alone	18.1
	Women (2)	48.5	Part-time contract	18.1	Married or in a marriage-like relationship	79.3	High school	5.9		Together with a partner	81.9
Age (in %) M = 37.29 SD = 10.202	15–24	1.7	Currently no contract	0.0	Divorced	0.8	Some college	68.8	Children at home (in %) M = 1.88 SD = 2.979	0	16.9
	25–34	43.5			Living with a partner	6.3	Bachelor's degree	19.8		1	36.6
	35–44	35.0			Widowed	0.4	Master's degree	1.7		2	34.9
	45–54	12.2								3	5.6
	55–65	5.9								4	0.8
	> 65	1.7								> = 5	5.2

All measurements are based on valid constructs adopted from past literature. T-LWC, S-LWC, and B-LWC are measured on scales developed by Carlson et al. (2000). We used the scales developed by Ahuja et al. (2007) to measure work exhaustion and the scale developed by Thatcher et al. (2002) to capture job satisfaction. Routine and innovative job performance is measured by the scale developed by Ali-Hassan et al. (2015) and IT telework environment is measured by the scale developed by Carillo et al. (2021). All constructs and their sources are shown in Appendix Table 8 (available at 10.1007/s12599-023-00800-3). We employ a variance-based structure equation modeling (SEM) approach using SmartPLS 3.3.3 (Ringle et al. 2015) to validate the research model.

## Validity, Reliability and Research Results

We ensure that our data are not subject to common method bias (CMB; see Appendix), and that the research model is valid and reliable, following generally accepted thresholds of validity and reliability.

### Measurement Model

To ensure that the measurement model we chose to test our hypotheses is valid and reliable, and because we measured all constructs with reflective indicators, we validated the measurement model in terms of content validity, indicator reliability, construct reliability, and discriminant validity (Bagozzi 1979).

*Content validity* We used items that have been used in previous research articles to ensure content validity (see Appendix Table 8) and discussed each item within our project team.

*Indicator reliability* This reflects the rate of the variance of an indicator that comes from the latent variables. Each value should be at least 0.707 to ensure that the indicators explain 50 percent or more of the variance (Carmines and Zeller 2008). All items which were below this threshold were removed from the model. Table S8 shows that this condition is fulfilled, and each loading has a significant level of at least 0.001.

*Construct reliability* We use composite reliability (CR) and average variance extracted (AVE) to determine construct reliability. CR should be at least 0.7. AVE has to be at least 0.5 (Fornell and Larcker 1981). Both criteria are fulfilled (see Table 3).

**Table 3:** Cross-correlations

		M	SD	AVE	CR	1	2	3	4	5	6	7	8	9	10	11	12	13	14
1	T-LWC	4.65	1.71	0.798	0.940	0.893													
2	S-LWC	4.47	1.75	0.817	0.930	0.808	0.904												
3	B-LWC	4.90	1.50	0.760	0.927	0.760	0.728	0.872											
4	Work exhaustion	4.66	1.74	0.839	0.954	0.683	0.765	0.680	0.916										
5	Job satisfaction	5.65	1.12	0.786	0.917	0.267	0.179	0.202	0.029	0.886									
6	Routine job performance	5.70	1.14	0.821	0.932	0.069	0.069	0.161	0.097	0.506	0.906								
7	Innovative job performance	4.08	1.61	0.804	0.961	− 0.218	− 0.272	− 0.335	− 0.325	0.132	0.045	0.897							
8	IT telework environment	5.52	1.07	0.722	0.838	0.253	0.237	0.305	0.147	0.538	0.570	0.014	0.850						
9	Telework experience	3.00	1.15	NA	NA	0.086	0.062	0.019	0.093	0.144	0.035	0.037	0.045	1.000					
10	Number of family members in household	3.49	1.27	NA	NA	0.215	0.183	0.165	0.183	0.093	0.050	0.012	0.048	0.037	NA				
11	Number of children at home	1.80	2.84	NA	NA	0.205	0.197	0.215	0.208	0.138	0.146	− 0.150	0.155	0.135	0.255	NA			
12	Hours for household chore and/or childcare per week	18.1	18.5	NA	NA	− 0.119	− 0.122	− 0.059	− 0.072	0.031	0.078	− 0.003	0.082	0.010	0.129	− 0.017	NA		
13	Telework extent	66.22	30.53	NA	NA	− 0.006	0.014	0.060	0.050	0.156	0.153	0.077	0.180	− 0.117	0.081	0.089	0.037	NA	
14	Age	37.29	10.20	NA	NA	− 0.105	− 0.096	− 0.042	0.033	0.039	0.082	− 0.004	0.061	0.119	− 0.041	0.010	− 0.000	− 0.050	NA
15	Gender	1.49	0.50	NA	NA	− 0.100	− 0.060	− 0.055	− 0.031	− 0.104	− 0.018	− 0.067	0.061	0.011	− 0.103	− 0.048	0.135	− 0.077	NA

Square root of AVE is listed on the diagonal of bivariate correlations

*NA*, not applicable; *AVE*, average variance extracted; *CR*, composite reliability;

*Discriminant validity* This reflects the extent to which items differ from other items (Campell and Fiske 1959). The square root of AVE should be higher than the corresponding construct correlations (Fornell and Larcker 1981; Hulland 1999). Table S8 shows that the square roots of the values are higher than the corresponding correlations between the constructs. Henseler et al. (2015) state that the Fornell-Larcker criterion does not detect a lack of discriminant validity in each case. Therefore, we also ensured that the heterotrait-monotrait (HTMT) criterion is fulfilled.

### Results

To validate the structural model, we use the coefficient of determination (R^2^) and the significance levels of the path coefficients (Chin 1998). Henseler et al. (2014) suggest using standardized root mean square residual (SRMR) for model fit. As the value of 0.04 is lower than the recommended value of 0.08, the model shows a good fit. The results in Table 4 show that T-LWC has a significant negative effect on routine job performance, such that H1c can be supported. No significant effect on work exhaustion and innovative job performance was found, such that H1a and H1d cannot be supported. Unexpectedly, T-LWC has a positive effect on job satisfaction, such that H1b cannot be supported. Regarding S-LWC, the results show a significant positive effect on work exhaustion, which supports H2a. The other relationships are not significant such that H2b, H2c, and H3d cannot be supported. Concentrating on B-LWC, the findings show a significant positive effect on work exhaustion and a negative effect on innovative job performance, supporting H3a and H3d. All other effects are not significant, such that H3b and H3c cannot be supported. Work exhaustion has a significant positive effect on routine job performance, such that H4a can be supported. The effect on innovative job performance is negatively significant, such that H4b can be supported. Job satisfaction significantly positively influences routine and innovative job performance, such that H5a and H5b can be supported. The IT telework environment has a significant effect on routine job performance, supporting H6a. No effect is found between the IT telework environment and innovative job performance, such that H6b cannot be supported. Among the control variables, the number of children in the household positively influences routine job performance and negatively influences innovative job performance. All other effects are insignificant. Work exhaustion can be explained by 61.9 percent, job satisfaction by 7.6 percent, routine job performance by 42.9 percent, and innovative job performance by 19.9 percent.

**Table 4:** Results structural model

	Work exhaustion	Job satisfaction	Routine job performance	Innovative job performance
T-LWC	0.068^NS^	0.339**	− 0.254*	0.131^NS^
S-LWC	0.537***	− 0.116^NS^	− 0.142^NS^	− 0.052^NS^
B-LWC	0.237**	0.028^NS^	0.084^NS^	− 0.321**
Work exhaustion			0.241*	− 0.166*
Job satisfaction			0.346***	0.134*
IT telework environment			0.417***	0.0466^NS^
*Controls*				
Telework experience^¥^			− 0.033^NS^	0.055^NS^
Number of family members in household^¥^			0.007^NS^	0.078^NS^
Number of children in household^¥^			0.047*	− 0.127*
Hours for household chore and/or childcare per week^¥^			0.013^NS^	− 0.043^NS^
Telework extent^¥^			− 0.003^NS^	0.083^NS^
Age^¥^			− 0.000^NS^	− 0.013^NS^
Gender			− 0.027^NS^	− 0.063^NS^
R^2^ (in %)	61.9	7.6	42.9	19.9
R^2^ (in %) without controls	64.4	6.4	41.0	14.8

Bold text highlights significant results; *NS*, p > 0.05; *R*^*2*^, coefficient of determination; *¥*, grand mean standardized; *T-LWC*, time-based personal life-work conflict; *S-LWC*, strain-based personal life-work conflict; *B-LWC*, behavior-based personal life-work conflict; *, p < 0.05; **, p < 0.01; ***, p < 0.001

### Post-hoc Analysis

The IT telework environment is an antecedent of job performance but also acts as a buffer between LWC and job performance and between job outcomes and job performance. For example, a good IT telework environment can reduce adverse effects resulting from the conflict between the personal life and work role, such that teleworkers demonstrate high performance even under high LWC. We conducted a moderation analysis to analyze these effects. The results in Table 5 demonstrate that the IT telework environment does not influence the effect of LWC, work exhaustion, and job satisfaction on routine job performance. In contrast, the findings show that the IT telework environment moderates the effect of job satisfaction, T-LWC, S-LWC, and B-LWC on innovative job performance. The IT telework environment does not moderate the relationship between work exhaustion and innovative job performance.

**Table 5:** Results of the moderation analysis

	Routine job performance		Innovative job performance	
	ß	95%CI	ß	95%CI
T-LWC × IT telework environment	0.010^NS^	[− 0.076; 0.145]	0.127*	[0.022; 0.218]
S-LWC × IT telework environment	0.017^NS^	[− 0.075; 0.140]	0.134*	[0.026; 0.226]
B-LWC × IT telework environment	− 0.002^NS^	[− 0.086; 0.118]	0.151**	[0.071; 0.232]
Work exhaustion × IT telework environment	− 0.074^NS^	[− 0.159; 0.028]	0.100^NS^	[− 0.026; 0.193]
Job satisfaction × IT telework environment	− 0.060^NS^	[− 0.123; 0.036]	0.109**	[0.050; 0.175]

Bold text highlights significant results; *NS*, p > 0.05; *R*^*2*^, coefficient of determination; *CI*, confidence interval; *T-LWC*, time-based personal life-work conflict; *S-LWC*, strain-based personal life-work conflict; *B-LWC*, behavior-based personal life-work conflict; *, p < 0.05, **, p < 0.01, ***, p < 0.001

**Fig. 3:**
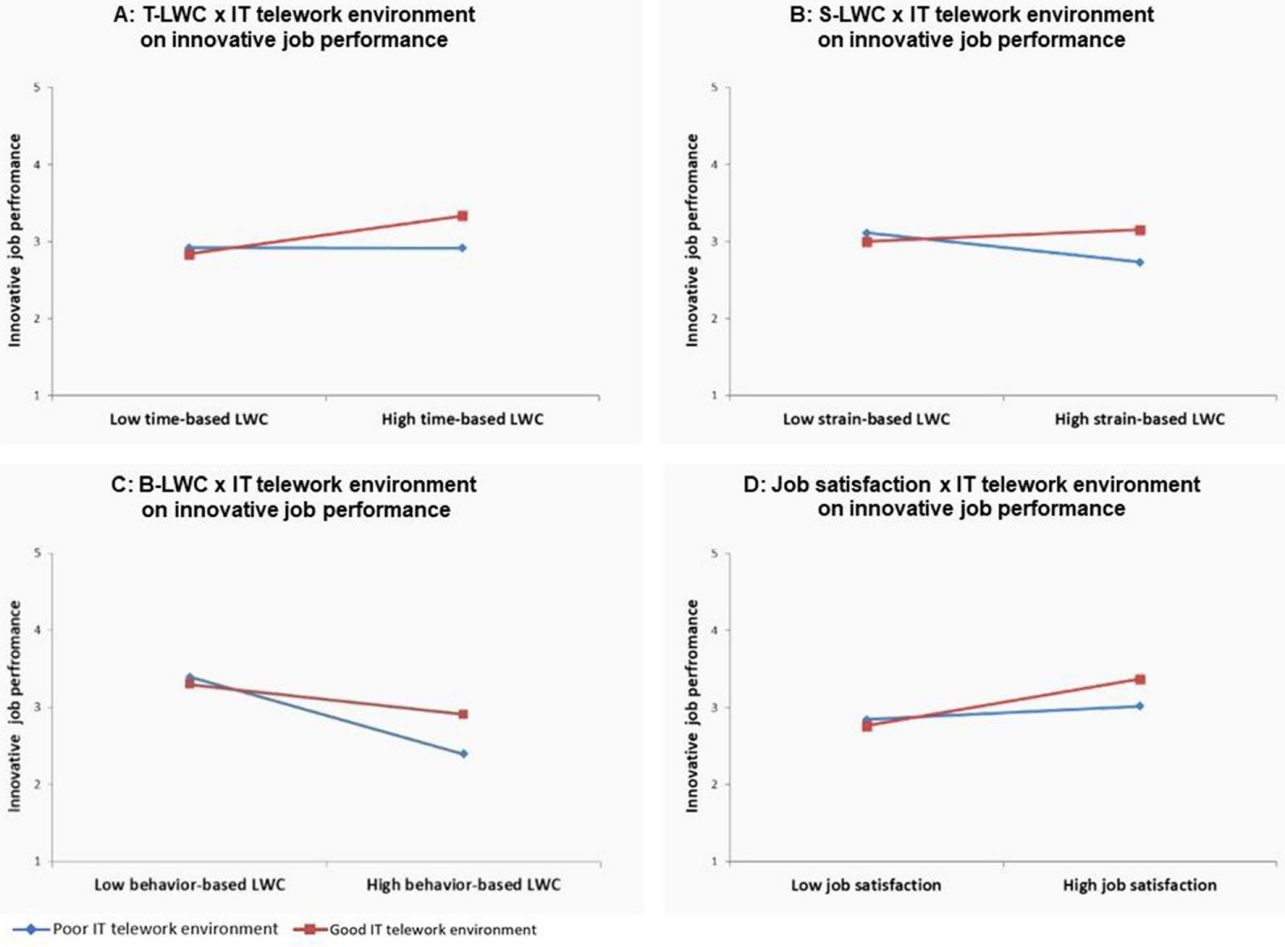
Significant moderation results

The significant moderation effects, shown in Fig. 3, indicate that teleworkers with a good IT telework environment under high T-LWC have a higher innovative job performance than teleworkers with a poor IT telework environment (Fig. 3A). Likewise, teleworkers with a good IT telework environment under high S-LWC have a higher innovative job performance than teleworkers with a poor IT telework environment (Fig. 3B). The moderation of the relationship between B-LWC and innovative job performance shows that teleworkers with a good IT telework environment under high B-LWC have a higher innovative job performance than teleworkers with a poor IT telework environment (Fig. 3C). The relationship between job satisfaction and innovative job performance is also moderated by the IT telework environment. Teleworkers with a good IT telework environment and who are highly satisfied with their job show an even better innovative job performance than those with a poor IT telework environment (Fig. 3D).

## Discussion, Implications, and Limitations

Alternative work arrangements, such as teleworking, have become more popular in the last years (Allen et al. 2015). Telework provides several advantages for employees, such as greater autonomy, flexibility, job satisfaction, and better performance (Kuruzovich et al. 2021; Bélanger 1999; Bélanger et al. 2001; Kossek et al. 2006). However, research also shows that teleworkers are vulnerable to personal life-work conflicts (LWC), because traditional boundaries between personal life roles and work roles blur when working and living in the same place (Delanoeije et al. 2019; Greer and Payne 2014). The Covid-19 pandemic accelerated the transition from traditional work arrangements (e.g., office work) to alternative arrangements such as teleworking. During the Covid-19 lockdowns, the government mandated that many organizations require or strongly encourage the employees in certain jobs to work from home to increase social distance. The lockdowns also changed the conditions under which teleworkers work. During the Covid-19 lockdowns, many teleworkers faced complex personal demands, such as supporting their children in remote learning and an ill-equipped telework environment as the result of a sudden and unplanned shift from office work to telework. If teleworker’s personal life role interferes with their work roles, especially if the IT telework environment is poor, undesirable job outcomes often result. Related extant IS literature only explores how the different LWC dimensions (i.e., time, strain, and behavior) result in undesired job outcomes – especially among the vulnerable group of teleworkers, even though a good IT telework environment is essential to achieve job performance when working at home. To fill this research gap, this study examines the influence of different dimensions of LWC (i.e., time, strain, behavior) on job outcomes (work exhaustion, job satisfaction, and routine and innovative job performance) and the mediating role of the IT telework environment on that influence. We consider the effect of LWC on each of the two main aspects of job performance – routine and innovative job performance – individually because, traditionally, teleworkers perform more routine tasks rather than knowledge tasks involving creativity and innovation when working from home (Pérez Pérez et al. 2002).

Our results show that S-LWC leads to work exhaustion, B-LWC leads to increased work exhaustion and lower innovative job performance, and T-LWC leads to lower routine job performance. Our results indicate that work exhaustion increases routine job performance but reduces innovative job performance. In contrast, job satisfaction has a significant positive effect on both routine and innovative job performance. The IT telework environment has a positive effect on routine job performance, meaning that good telework IT conditions, including sufficient hard- and software and access to applications, facilitate accomplishing routine tasks.

Our post-hoc analysis further reveals that the IT telework environment moderates the relationship between LWC and innovative job performance such that teleworkers with high T-LWC, S-LWC, and B-LWC who have a good IT telework environment demonstrate better innovative job performance than those who have a poor IT telework environment. The IT telework environment also moderates the relationship between job satisfaction and innovative job performance, such that teleworkers who are highly satisfied in their job and who have a good IT telework environment demonstrate better innovative job performance than those who have a poor IT telework environment. Contrary to our hypotheses, we also found a positive effect between T-LWC and job satisfaction. One explanation might be that teleworkers who do not have enough time for their work roles because of responsibilities in their personal life roles feel that they are needed in their job or might perceive the time urgency as challenging, which leads to positive responses (Benlian 2020) such as job satisfaction. These findings contain several theoretical and practical implications, which we discuss subsequently.

### Implications for Research

This study contributes to research in four primary ways. First, our results demonstrate that the IT telework environment is essential to achieve routine and innovative job performance in the context of telework. During the Covid-19 lockdowns, employees were forced to work remotely from home suddenly and unexpectedly and many were technically ill-equipped, working on small laptops with insufficient access to organizational software and poor internet connections. Our findings build upon extant research findings that adequate IS applications and communication technologies increase teleworkers’ productivity (Bélanger et al. 2001) and that IS quality affected productivity among teleworkers, as was evident at the beginning of the Covid-19 pandemic (Kuruzovich et al. 2021; Wang et al. 2020). Our results align well with previous results, showing that a good IT telework environment increases routine job performance, but also extend previous results. Prior to this study, there was no evidence that the IT telework environment impacts innovative job performance among teleworkers. By analyzing how different types of personal life-work conflicts affect routine and innovative job performance individually (Ali-Hassan et al. 2015; Janssen and Van Yperen 2004; Katz 1964), we provide empirical evidence that the IT telework environment does not directly affect innovative job performance but rather mitigates adverse personal situations that adversely affect innovative job performance. Our results show that teleworkers with high interference between the personal life and work role and a good IT telework environment demonstrate higher innovative job performance than those with a poor IT telework environment. These findings will be of broad use to research as we demonstrate that teleworkers are more capable of accomplishing routine and innovative tasks in a good IT telework environment.

Second, by analyzing the effects of LWC on routine and innovative job performance among teleworkers individually, we extend telework research analyzing simply the overall performance of teleworkers (Bélanger et al. 2001; Wang et al. 2020; Kuruzovich et al. 2021). Traditionally, teleworkers have focused primarily on routine tasks, rather than on knowledge development, creativity and innovation (Pérez Pérez et al. 2002). In the (post)-Covid-19 pandemic, instigated by mandated teleworking during the Covid-19 lockdowns, teleworkers focus on the full range of work tasks. We build on previous knowledge about teleworkers’ job performance (van der Meulen et al. 2019; Kuruzovich et al. 2021; Wang et al. 2020) by considering routine and innovative job performance individually, thus revealing different antecedents: Routine job performance is negatively influenced by T-LWC but positively influenced by work exhaustion, job satisfaction, and the IT telework environment; Innovative job performance is negatively influenced by B-LWC and work exhaustion and only positively affected by job satisfaction. These new insights will guide organizations in helping teleworkers achieve better routine and innovative performance and develop specific countermeasures to reduce negative antecedents.

Moreover, we extend extant research revealing a negative effect of LWC on job performance during Covid-19 restrictions (Wang et al. 2020) by differentiating between S-LWC, T-LWC, and B-LWC. Our results show a negative effect of T-LWC on routine job performance and of B-LWC on innovative job performance. While our results underscore that teleworkers experiencing LWC perform better at routine job tasks than at innovative job tasks, our results also indicate how strong innovative job performance can be achieved (see Table 3). In summary, differentiating between routine and innovative job performance provides a deeper understanding of the factors affecting strong telework job performance.

Third, our study focuses on LWC, whereas most extant research concentrates on WLC (e.g., Ahuja et al. 2007; Sarker et al. 2018; Weinert et al. 2016; Sarker et al. 2010; see Table 1) and hence neglects detrimental spillover effects from a person’s personal life into the work life. By differentiating between T-LWC, S-LWC, and B-LWC, we provide a far more granular understanding of how various types of personal life-work conflicts affect teleworkers’ job performance. For example, whereas addiction research (Venkatesh et al. 2019) shows that LWC has a negative effect on job satisfaction, we reveal a positive effect between T-LWC and job satisfaction. Furthermore, prior research into the effect of WLC on job performance reveals no significant advantage of distinguishing WLC and job performance into their fundamental parts (Sarker et al. 2018). In contrast, our findings show that T-LWC lowers routine job performance and B-LWC lowers innovative job performance. By focusing on LWC dimensions, we are able to compare the effects of LWC with WLC from past research (Weinert et al. 2016). For example, past results show that T-WLC and S-WLC have a significant effect on work exhaustion, while our results show that S-LWC and B-LWC influence work exhaustion while T-LWC has no significant effect. Our findings contribute to our understanding of role conflicts, showing that, in addition to WLC, LWC dimensions also impact work exhaustion, job satisfaction, and job performance in the context of telework.

Fourth, the interference between personal life roles and work roles has been studied in various technology-related contexts, such as among IT professionals (Armstrong et al. 2015; Ahuja et al. 2007; Sarker et al. 2018, 2010), individuals stressed by IT (Ayyagari et al. 2011), and IT addictive individuals (Venkatesh et al. 2019). Our study adds to this body of knowledge by focusing on the increasingly important group of teleworkers vulnerable to role conflicts (Greer and Payne 2014; Delanoeije et al. 2019), especially in the (post)-Covid-19 pandemic. Before the Covid-19 crisis, telework was expected to grow as employees looked for alternative ways to organize their work and personal lives utilizing ever-increasing digitalization. Teleworkers working at home face specific challenges because working and living at the same place blurs the boundaries between personal life roles and work roles and creates conflicts that can positively or negatively affect their job performance. The unexpected and unplanned spike in teleworking during the Covid-19 lockdowns provides a unique opportunity to research the difficult conflicts teleworkers face, particularly in the (post)-Covid-19 pandemic. As discussed in detail above, our results provide new insights into the complex effects of various dimensions of LWC on both routine and innovative job performance, which will help practitioners design better telework arrangements.

### Implications for Practice

The Covid-19 pandemic has catalyzed the increase of alternative work arrangements such as teleworking. The share of full- or part-time employees or self-employed individuals in the EU with a contract with an organization who telework at least some of the time increased from 5 percent in 2019 to a peak of 40 percent (Eurofound 2020). Given the generally positive response of employees and employers to the unexpected and unplanned telework experience during the Covid-19 lockdowns (Milasi et al. 2020; Eurofound 2020), telework is likely to remain common in the (post)-Covid-19 pandemic. However, more extensive telework means that the blurry boundaries between work and personal life will increasingly result in conflicts and threaten job outcomes. LWC among teleworkers and its consequences demonstrate that telework, besides its positive effects such as more autonomy and flexibility, also has a negative side which should be considered when employees aspire to work from home. Practitioners are discussing the new skills needed to manage rapid and frequent transitions between roles of home and work, which make employees anxious (Relaxnews 2021). Hence, organizations can learn from the present research that the IT telework environment is essential for the routine and innovative job performance of teleworkers in the context of LWC. Organizations must ensure that teleworkers are well equipped with IT and have sufficient access to IS applications. The IT telework environment has a direct effect on routine work performance, enabling teleworkers to accomplish their daily tasks more effectively and enabling teleworkers to mitigate the effects LWC. Our results show that teleworkers perceiving high LWS who have a good IT environment demonstrate better innovative job performance than those who have a poor IT environment. The negative effect of interference between personal life roles and work roles on job outcomes among teleworkers we demonstrate underscores the importance of ensuring that especially teleworkers have the “right to switch off” to minimize the risk of physical and emotional exhaustion. To design telework arrangements effectively, organizations must understand the role conflicts that can arise when teleworkers live and work in the same place. Based on our findings, organizations should be especially aware of the negative effects of T-LWC and B-LWC on both routine and innovative job performance and develop countermeasures such as training for their teleworkers to reduce the interference their private life roles and their work roles.

### Limitation and Future Research

As with all research, our study is limited in several ways that indicate the need for future research. First, to fill an observed research gap, we focused solely on the interference between personal life roles with work roles, and thus neglected the interference between work roles and personal life roles, which has been investigated in previous literature (e.g., Sarker et al. 2010, 2018; Ahuja et al. 2007; Ayyagari et al. 2011). Future research should investigate how various types of WLC affect personal life outcomes during the (post)-Covid-19 pandemic. Second, we focus on the organizationally relevant outcomes of work exhaustion, job satisfaction, and job performance. Future research should also consider organizational commitment and turnover intention, as well as personal outcomes such as personal and family satisfaction, potentially considering the question of surveillance (Manley and Williams 2019) in teleworking. Besides, the autonomy of employees is also an essential factor when teleworking during the Covid-19 pandemic (Wang et al. 2020). Past literature reveals an autonomy paradox claiming that IT such as mobile email devices increases and decreases the autonomy of professionals (Mazmanian et al. 2013). Further research should investigate these counteractive effects of autonomy and IT use in the context of teleworking. In the Covid-19 pandemic, future research might also consider additional confounding variables such as personal illness, illness of family members, or health concerns. Third, based on our finding that a good IT telework environment facilitates strong routine and innovative job performance despite LWC, future research should investigate the specific relevant characteristics of a good IT telework environment. Fourth, we build on findings from extant telework research (Greer and Payne 2014) that teleworkers are more vulnerable to role conflict than office workers because the at-home environment blurs role boundaries. Future research should compare teleworkers with non-teleworkers and/or employees who are teleworking during one timeframe and not teleworking during a second timeframe to understand more holistically the dimensions of LWC and their effects on job outcomes. Thereby, it should be considered that all employees have no telework experience. Another fruitful avenue might be focusing on the quick switch between office work and telework. Fifth, our findings point to the fact that rapid and frequent transitions between personal life roles and work roles affect job outcomes, but future research should investigate the potential positive and negative effects of teleworkers switching frequently and rapidly between personal life roles and job roles and thereby carrying over behavior from personal life roles to job roles in the (post)-Covid-19 pandemic. Sixth, in our study, we included part-time and full-time employees and self-employed individuals who have a contract with an organization and telework at least some of the time and whose work requires them to use IT to some degree. Some extant research only considers employees who telework 100 percent from home (e.g., Lindström et al. 1997). Future research might investigate the effects of the need of IT to accomplish the working tasks and the extent of teleworking on the different job outcomes. Also, in our study, we surveyed the affiliation of the teleworkers to the organizational sectors and working fields (Appendix Table 7) but do not control for different job types. Future research should differentiate among different job types and analyzes their effect on teleworking, LWC, and job outcomes. Future research into the effect of LWC on teleworkers’ job outcomes should distinguish among more narrowly defined groups and control groups of teleworkers, e.g., share of telework vs. office work, full-time vs. part-time employees, job type/intensity of dependence on information and communication technologies, organizational sectors and fields of work. Seventh, our study relies on self-reported job performance. Future research should use additional measures of job performance, such as supervisor evaluation. Eighth, our study does not consider leadership approaches (Fernet et al. 2015). Future research should build on extant telework research comparing the effectiveness of various leadership approaches, including some developed in the (post)-Covid-19 pandemic (e.g., Fischer and Grunnet 2021), perhaps using advanced analysis techniques such as hierarchical linear modeling. Finally, our sample was collected at a single time and in a single geographical location during the Covid-19 pandemic. Future research might test our results during a different phase of the (post)-Covid-19 pandemic and in different geographical/cultural settings.

## Conclusion

Teleworkers are more vulnerable to interference between their personal life roles and their work roles, resulting in LWC. Particularly during the Covid-19 lockdowns, when governments mandated that organizations required or strongly encouraged many of their employees to make the sudden and unexpected shift from office work to telework, the risk and frequency of LWC increased dramatically. During the Covid-19 lockdowns, telework conditions were often substandard and challenging. Teleworkers faced difficult personal life situations and often worked in an ill-equipped telework IT environment. Many teleworkers lived and worked in the same space as children, spouses, partners and/or roommates. This unique telework situation created a fruitful opportunity to investigate the effects of different dimensions of LCW (e.g., time, strain, behavior) on work exhaustion, job satisfaction, and routine and innovative job performance among teleworkers, and how the IT telework environment influences job performance among teleworkers. Our findings show that LWC has adverse effects on job outcomes and different antecedents of job performance. Routine job performance is only negatively influenced by T-LWC, while work exhaustion, job satisfaction, and the IT telework environment positively affect routine job performance. In contrast, innovative job performance is negatively affected by B-LWC and work exhaustion and positively affected by job satisfaction. The IT telework environment has no direct effect on innovative job performance, but it mitigates the adverse effects of LWC on innovative job performance.

## Supplementary Information

Below is the link to the electronic supplementary material.

Supplementary file1 (PDF 87 KB)

## Footnotes

1 In this study, we use the terms personal life-work conflict (LWC) and work-personal life conflict (WLC). However, there are numerous terms which are used largely interchangeably, such as work-life or life-work conflict (Sarker et al. 2018; Benlian 2020), work-home or home-work conflict (Ayyagari et al. 2011, Ahuja et al. 2007), work-family or family-work conflict (Turel et al. 2011; Venkatesh et al. 2019).

2 Past literature demonstrates that there are many concepts, such as telecommuting, mobile work, virtual work, and work at home. The terms are defined in many ways and used interchangeably (Lindström et al. 1997). We focus on at-home telework because this concept best explains the situation during the Covid 19 closures and because there is a large body of literature.
