# Supplementary material for: Teleworking in the Covid-19 Pandemic: The Effects of Life-Work Conflict on Job Outcomes and the Role of the IT Telework Environment
Source: Bus Inf Syst Eng. 2023 Mar 17:1–20. Online ahead of print. doi: 10.1007/s12599-023-00800-3 (PMC10023025; doi:10.1007/s12599-023-00800-3)
Supplement: Supplementary file 1 — Supplementary file1 (PDF 87 KB) [file 12599_2023_800_MOESM1_ESM.pdf]

Teleworking in the Covid-19 Pandemic –

The Effects of Life-Work Conflict on Job Outcomes and the Role of the IT Telework Environment

Christoph Weinert, Tim Weitzel

Business & Information Systems Engineering (2023)

Appendix (available online via http://link.springer.com)

Common Method Bias: Perceived and subjective measures are used to capture teleworkers' responses to a given situation. A potential issue with subjective measures is common method bias (Podsakoff et al. 2003). To evaluate the extent of CMB, we utilize Harman's single factor test (Harman 1976). The results show that one factor explains 36.9% of the variance, which is not the majority, such that we conclude that CMB is of no great concern. Furthermore, we follow the procedure suggested by Williams et al. (2003), during which an additional factor is entered into the PLS model, which contains each indicator of the original model. The remaining factors are transformed into single-item constructs, and the ratio of R^2 with the CMB factor is compared with the R^2 without the CMB factor. The CMB factor explains an average R^2 of 0.018 so that a ratio of 1:1356 is received. In addition, most factor loadings are not significant. By comparing this ratio with the ratio used by prior researchers (Liang et al. 2007), we can state that no influence of CMB is observed, despite several flaws in this method (Chin et al. 2012). Besides, we test for multicollinearity. As indicated by the variance inflation factors (VIF), each VIF value is lower than the recommended maximum, which is 5 (Rogerson 2015). We also checked the correlation matrix for extremely high correlations ($r > 0.90$) because such high correlations are an indicator of CMB (Pavlou et al. 2007). Our correlation matrix indicates no such high correlations.

Table 7 Sector of the organizations and working fields

**Sector of the organizations**		**Working fields**	
Construction industry	3.4	Architecture	1.7
Electrical engineering, information technology (IT), data processing	21.1	Training or continuing education of third parties	1.7
Energy and water supply	0.8	Consulting	1.7
Food industry and tobacco processing, gastronomy	2.5	Design	2.5
Education and training	8.9	Purchasing/materials management	2.5
Vehicle construction	1.3	Finance/accounting/controlling	14.8
Financial Services	16.9	Research and development (R&D)	1.7
Research and development (R&D)	1.7	IT/EDP	28.7
Health, veterinary and social work	7.2	Journalism/editorial activities	1.7
Land and housing	1.7	Marketing	5.9
Trade	1.3	Medical professions	4.2
Handicraft	1.3	Scientific professions	1.3
Mechanical Engineering	2.5	Organization/Administration	3.0
Public administration, social security	1.3	Human Resources	3.0
Transport of passengers and goods, mail	0.8	Legal system	0.8
Other manufacturing industry	4.2	Technical professions/engineering	15.6
Telecommunication services, communication	18.6	Transportation	1.3
Textiles and clothing, leather	0.4	Distribution/Sales	2.5
Economic services (consulting)	0.4	Others	5.5
Others	3.8		

Table 8 Measurement items

Construct	Items	LD
**Time-based LWC (Carlson et al. 2000)** Cronbachs α = 0.915	My personal personal life takes up time that I'd like to spend at work.	0.874
	The time I spend on family responsibilities often interfere with my work responsibilities.	0.924
	I find myself making family related phone calls or running personal errands during work time.	0.899
	I have to miss work activities due to the amount of time I must spend on family responsibilities.	0.885
**Strain-based LWC (Carlson et al. 2000)** Cronbachs α = 0.887	I'm often too tired at work because of the things I have to do at home.	0.874
	Due to stress at home, I am often preoccupied with family matters at work.	0.891
	Tension and anxiety from my family personal life often weakens my ability to do my job.	0.945
**Behavior-based LWC (Carlson et al. 2000)** Cronbachs α = 0.895	Behavior that is effective and necessary for me at home would be counterproductive at work.	0.850
	The problem solving behavior that works for me at home does not seem to be as useful at work.	0.898
	In order for me to succeed at work, I must be a different person than I can be at home.	0.871
	The behaviors I use to respond to interpersonal problems at work perform better at home than at work.	0.868
**Work exhaustion (Ahuja et al. 2007)** Cronbachs α = 0.936	I feel emotionally drained from my work.	0.921
	I feel used up at the end of the work day.	0.918
	I feel fatigued when I get up in the morning and have to face another day on the job.	0.910
	I feel burned out from my work.	0.914
**Job satisfaction (Thatcher et al. 2002)** Cronbachs α =0.864	Overall, I am satisfied with my job.	0.886
	I am satisfied with the way I work at the moment.	0.877
	I am satisfied with the important aspects of my job.	0.896
**Routine job performance (Ali-Hassan et al. 2015)** Cronbachs α = 0.891	To what extent do you agree or disagree with the following?	
	I always complete the duties specified in my job description.	0.888
	I always meet all the formal performance requirements of my job.	0.919
	I always fulfill all responsibilities required by my job.	0.912
	I often fail to perform essential duties.(dropped)	
**Innovative job performance (Ali-Hassan et al. 2015)** Scale**:** 1 = never to 7 = every time Cronbachs α = 0.951	How often do you perform the following work activities?	
	Create new ideas for improvements	0.856
	Mobilize support for innovative ideas	0.911
	Search out novel working methods	0.900
	Transform innovative ideas into useful applications	0.910
	Generate original solutions to problems	0.903
	Introduce innovative ideas	0.898
**IT telework environment (Carillo et al. 2021)** Cronbachs α = 0.615	I have a good IT telework environment to work from home.	0.846
	I have satisfactory access to professional IT tools from home (professional software, messaging, shared files, video conference...).	0.853
**Telework experience (Carillo et al. 2021)** Scale: 1 = 0 years to 6 = longer than 10 years	I had worked in telework for my employer before the current situation of the Covid-19 virus.	NA
**Telework extent** (=hours per week remotely/hours per week in total)	How many hours per week do you work?	NA
	How many hours per week do you work remotely from home (telework)?	NA
**Number of family members in household**	How many family members live in your household?	NA
**Number of children at home**	How many of your children live at home (in the same apartment or house as you)?	NA
**Hours for household chore and/or childcare per week**	The number of hours spent per week on family- and home-related duties such as housework and childcare:	NA
Note: Unless otherwise stated, items are assessed on a 7-point Likert scale (1= strongly agree to 7 strongly disagree); LD = factor loadings		
